# PARP7 Suppresses Radiation-induced Necroptosis and Abscopal Immunity

**DOI:** 10.21203/rs.3.rs-7881707/v1

**Published:** 2025-11-07

**Authors:** Gaorav Gupta, Anna Goddard, Sierra McDonald, Lynn Lerner, Maxwell Finkelstein, Qinhong Wang, Faeze Gharibpoor, Min-Guk Cho, Simon Ellington, Matthew Sutcliffe, Kevin Raynard Mott, William Green, Amber Gomez, Steven Vensko, J. Justin Milner, Benjamin Vincent, Charles Perou

**Affiliations:** University of North Carolina at Chapel Hill; University of North Carolina at Chapel Hill; University of North Carolina at Chapel Hill; University of North Carolina at Chapel Hill; University of North Carolina at Chapel Hill; University of North Carolina at Chapel Hill; University of North Carolina at Chapel Hill; University of North Carolina at Chapel Hill; University of North Carolina at Chapel Hill; University of North Carolina at Chapel Hill; University of North Carolina School of Medicine; University of North Carolina at Chapel Hill; University of North Carolina at Chapel Hill; University of North Carolina at Chapel Hill; University of North Carolina; UNC-Chapel Hill; University of North Carolina at Chapel Hill

## Abstract

The abscopal effect, in which local radiotherapy (RT) drives regression of distant tumors, remains unpredictable and mechanistically elusive. Using a panel of p53-null murine breast cancer models, we identified tumor-intrinsic determinants of abscopal competence to RT and immune checkpoint inhibitors (ICI). Abscopal-competent tumors exhibited heightened type I interferon stimulated gene (ISG) expression, induction of the necroptosis mediator ZBP1, and recruitment of antigen-presenting cells (APCs) and effector T cells in distant tumors. Transcriptomic analyses revealed PARP7 as a tumor-intrinsic suppressor of RT-induced ISGs and necroptosis. Pharmacologic PARP7 inhibition amplified RT-driven ISGs and ZBP1-dependent necroptosis. *In vivo*, PARP7 blockade combined with RT + ICI conferred abscopal competency to resistant tumors, improving distant tumor control, systemic immune activation, and survival. Notably, ZBP1 loss abrogated these effects, preventing APC recruitment and T cell priming. These findings establish PARP7 and necroptosis as opposing regulators of abscopal responses and nominate PARP7 inhibition as a strategy to overcome RT + ICI resistance.

## INTRODUCTION

Radiotherapy (RT) exerts both stimulatory and suppressive effects on anti-tumor immunity^1–3^. In combination with immune checkpoint inhibitors (ICI), radioimmunotherapy can help overcome ICI resistance by enhancing tumor antigen presentation and effector T cell activity^4–7^. Important insights have emerged regarding the benefits of hypofractionated RT^8–10^, concurrent sequencing with ICI^4,11–13^, and regional lymph node sparing^14–16^ in enabling robust anti-tumor immune responses. Nonetheless, regression of distant non-irradiated lesions after RT + ICI—termed the “abscopal” effect—remains rare in clinical practice^17,18^. While some trials of RT + ICI combinations have reported encouraging signals, most have not met expectations^11,19–23^.

The heterogeneity, rarity, and unpredictability of abscopal responses underscore a critical knowledge gap in understanding the determinants of RT + ICI-induced immunity across clinically relevant, ICI-resistant cancer models. Systematic comparisons of the immunological consequences of RT + ICI in abscopal competent versus incompetent models have not previously been reported. Because abscopal immunity requires coordinated effects in both irradiated and distant non-irradiated tumors, along with essential crosstalk with regional lymph nodes^24^, the mechanisms that either enable or restrict these processes remain poorly defined. Elucidating them will be crucial not only for refining patient selection in RT + ICI trials, but also for identifying targetable strategies to broaden the therapeutic potential of radioimmunotherapy.

Modulating DNA damage responses with DNA repair inhibitors is a potential strategy to enhance anti-tumor immunity induced by RT^25–27^. PARP1/2 inhibitors, for instance, can activate cGAS/STING-mediated type I interferon signaling not only in BRCA-deficient cancers but also in non-BRCA mutant tumors treated with RT, thereby augmenting responses to RT + ICI^28–30^. More broadly, the PARP family of poly-ADP ribosyl polymerases constitutes a druggable class of enzymes with diverse biological functions. Certain members, such as PARP7 (gene name *TIPARP*), are implicated primarily in immune suppression and ICI resistance^31,32^, yet their role in shaping abscopal responses to RT + ICI has not been defined.

The cytotoxic effects of RT are accompanied by the release of damage-associated molecular patterns (e.g., calreticulin, ATP, and HMGB1) and the induction of context-specific cell death programs, including apoptosis, pyroptosis, ferroptosis, and necroptosis^33–35^. Whereas apoptosis is generally immunologically silent^36^, necroptosis—driven by ZBP1- and MLKL-dependent membrane permeabilization—is highly immunostimulatory^37^. Ectopic induction of necroptosis can overcome ICI resistance through enhanced antigen cross-presentation by BATF3 + type I conventional dendritic cells (cDC1) and activation of CD8^+^ and CD4^+^ effector T cells^37–39^. Collectively, these findings suggest that the capacity to induce necroptosis in response to RT may be an important determinant of abscopal immunity. However, a direct causal role for tumor-intrinsic necroptosis in abscopal responses to radioimmunotherapy has not been established, nor have therapeutic strategies to enhance this pathway been explored.

Here, we systematically evaluated abscopal responses to radioimmunotherapy across a panel of p53-deficient syngeneic triple negative breast cancer (TNBC) models that capture the molecular heterogeneity of human disease^40,41^. Using transcriptomic and immune profiling approaches, we identify tumor-intrinsic features that distinguish abscopal-competent from incompetent tumors, including type I interferon signaling and ZBP1-dependent necroptosis. We further demonstrate that PARP7 acts as a suppressor of these pathways and that its inhibition enhances abscopal immunity by promoting tumor-intrinsic necroptosis.

## RESULTS

### Quantifying the Abscopal Response in Immune Cold Breast Cancer Models

**Figure 1:**
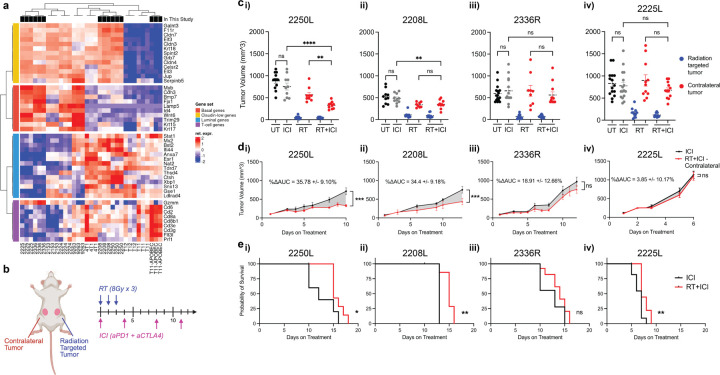
Models and treatment design to assess abscopal response. (a) PAM50 classification of p53-null BALB/c mammary tumor syngeneic models with those used in this study highlighted in black: luminal-like (2250L, 2208L), basal (2336R, 2225L), and claudin-low (T11-APOBEC). (b) Experimental framework and treatment timeline. p53-null BALB/c mice were bilaterally injected with tumor cells (2250L, 2208L, 2336R, or 2225L) and randomized at ~6 mm tumor size to no treatment (untreated), hypofractionated radiotherapy (RT; 8 Gy × 3), dual immune checkpoint blockade (ICI; anti–PD-1 10 mg/kg + anti–CTLA-4 5 mg/kg twice weekly), or combined RT+ICI. The irradiated tumor was designated targeted; the contralateral unirradiated tumor assessed abscopal effects. (c) Static tumor volume on day 10 of treatment for each model: (i) 2250L, (ii) 2208L, (iii) 2336R, and (iv) 2225L. Fisher’s LSD test, *p<0.05, **p<0.01, ***p<0.001, ****p<0.0001. (d) Delta area under the curve analysis (ΔAUC) of ICI vs. contralateral RT+ICI tumors; unpaired t-test, (i) p=0.0003, (ii) p=0.0006, (iii) p=0.1426, (iv) p=0.7067. Dates of comparison determined by date at which half the control group reached tumor burden. (e) Kaplan-Meier survival of ICI vs. RT+ICI for each tumor model, (i) 2250L, p=0.0190, (ii) 2208L, p=0.0051, (iii) 2336R, p=0.1010, (iv) 2225L, p=0.0083.

To evaluate the impact of TNBC molecular heterogeneity on the abscopal response, we selected four p53-null BALB/c mammary tumor allograft models spanning basal (2336R, 2225L) and luminal-like (2250L, 2208L) subtypes^42,43^. These tumors are largely immune cold, as reflected by low T cell signatures compared to the immunologically hot APOBEC mutagenized claudin-low TNBC model, T11-APOBEC (Fig. 1a)^44^. Notably, the luminal-like models (2250L, 2208L) exhibited modestly higher T cell infiltration at baseline than the basal lines (Fig. 1a). All four models harbored low tumor mutational burden and predicted neoantigen load (Fig. S1a-b), suggesting limited intrinsic immunogenicity and a low likelihood of ICI responsiveness.

To assess treatment-induced abscopal effects, mice received bilateral orthotopic injections of tumor cells and, upon reaching ~ 6mm in size, were randomized to receive: no treatment, hypofractionated RT (8 Gy × 3) to a single tumor, dual immune checkpoint inhibitor (ICI: anti–PD-1 and anti–CTLA-4 (Cytotoxic T-Lymphocyte Associated protein 4)), or combined radioimmunotherapy (RT + ICI) (Fig. 1b). The irradiated tumor was designated the “targeted” tumor; the unirradiated contralateral tumor served to evaluate the abscopal response.

**Figure 2:**
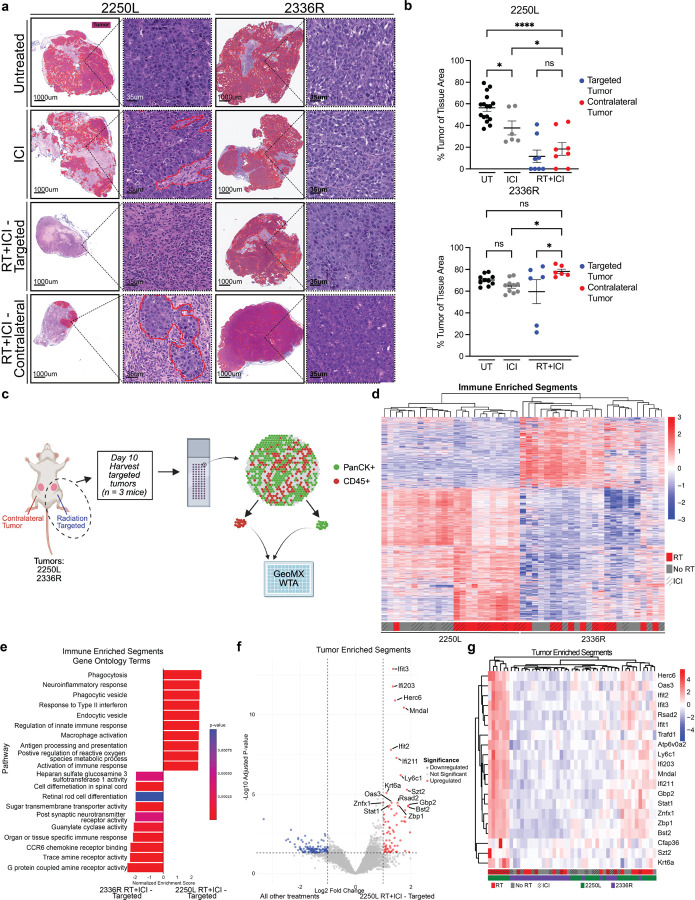
Molecular correlates of the abscopal response. p53-null BALB/c mice bearing 2250L or 2336R tumors were harvested on day 10 of treatment and fixed for hematoxylin and eosin (H&E) analysis and spatial transcriptomics. (a) Representative H&E images of tumors from untreated, ICI, RT+ICI–targeted and RT+ICI–contralateral 2250L and 2336R mice. Epithelial tumor areas are shown as red overlays. Scale bar = 35mm. (b) Quantification of tumor percentage on H&E in 2250L (upper) and 2336R (lower). Fisher’s LSD test, * p<0.05, **p<0.01, ***p<0.001, ****p<0.0001. (c-g) Spatial transcriptomics performed on untreated (no RT) and targeted tumors receiving either RT alone, ICI alone, or RT+ICI for both models (2250L or 2336R). (c) Spatial transcriptomic workflow: targeted tumors were arranged in a tissue microarray, stained for PanCK and CD45, and tumor- and immune-enriched areas of interest were collected and profiled. (d) Unsupervised hierarchical clustering of differentially expressed genes (DEGs) in immune-enriched segments for both 2250L and 2336R. (e) Gene set enrichment analysis of immune-enriched segments comparing 2250L RT+ICI–targeted and 2336R RT+ICI–targeted tumors using Gene Ontology terms. (f) Volcano plot of DEGs in 2250L RT+ICI–targeted vs all other treatments (2250L and 2336R grouped together) in tumor-enriched segments. (g) Unsupervised hierarchical clustering of the top 20 DEGs distinguishing 2250L RT+ICI–targeted tumors from all other treatments (2250L and 2336R grouped together) in tumor-enriched segments.

We defined abscopal response using three criteria: (1) reduced contralateral tumor volume relative to untreated and ICI-only groups at a landmark timepoint (day 10 from treatment initiation) (Fig. 1c); (2) significant contralateral tumor growth inhibition over time compared to ICI-only treatment, quantified by delta area under the curve analysis (ΔAUC) (Fig. 1d); and (3) improved overall survival assessed using the Kaplan-Meier method (Fig. 1e). Models meeting all three criteria were classified as “abscopal competent.”

All four models were resistant to ICI monotherapy based on ΔAUC and landmark growth metrics, when compared to untreated tumors (Fig. 1c, S1c–d). While targeted tumors in all models responded similarly to radiation alone and RT + ICI, only 2250L and 2208L treated with RT + ICI showed significant contralateral tumor growth inhibition by both landmark and ΔAUC analyses (Fig. 1c–d). No significant abscopal response was observed in models 2336R or 2225L (Fig. 1c–d iii–iv). Despite initial response, abscopal effects in 2250L and 2208L were transient and ultimately lost as contralateral tumors progressed (Fig. S1d–f). Nonetheless, RT + ICI significantly prolonged survival in abscopal models, while the benefit was minimal or absent in non-abscopal lines (Fig. 1e, S1g). We thus defined 2250L and 2208L as abscopal-competent, and 2336R and 2225L as non-abscopal models.

### Molecular Correlates of the Abscopal Response

The differences in RT + ICI abscopal responses were associated with distinct histopathological phenotypes. Despite irradiated “targeted” tumors exhibiting comparable tumor sizes across all 4 models, the percentage of the tumor comprised with viable tumor cells histopathologically was markedly reduced in abscopal competent model 2250L compared to non-abscopal 2336R, which was also mirrored in contralateral tumors (Fig. 2a–b).

We hypothesized that differences in radiation-induced tumor cell signaling to the local immune microenvironment may underlie abscopal competency. To dissect this, we performed spatial transcriptomic profiling (GeoMx WTA) of tumor cells and tumor-adjacent immune cells from tumors from each treatment group in the 2250L (abscopal competent) and 2336R (non-abscopal) models. Tumor and immune compartments in radiation-targeted tumors with or without ICI were defined via PanCK and CD45 co-staining, respectively, with tumor-immune interface regions of interest (ROIs) segmented into tumor- and immune-enriched areas of interest (AOIs) (Fig. 2c, S2a-c).

Unsupervised hierarchical clustering of immune AOI transcriptomes revealed that immune microenvironments segregated by tumor type and, in 2250L, further subclustered by radiation treatment. A similar effect was not observed after RT in 2336R immune-enriched AOIs (Fig. 2d, S2d), suggesting that differences in radiation-induced immune remodeling may underlie abscopal competency. Gene set enrichment analysis (GSEA) of immune AOIs from 2250L versus 2336R after RT + ICI treatment revealed upregulation of phagocytosis, IFN-γ response, macrophage activation, antigen presentation, and innate immune response gene sets in the abscopal competent 2250L model (Fig. 2e, S2e-f). These observations suggest that recruitment and activation of phagocytic myeloid antigen presenting cells (APC) in RT + ICI-treated tumors is a characteristic feature of abscopal responses.

GSEA of tumor cell-enriched (i.e., panCK positive) AOIs in RT + ICI-treated samples was notable for elevated type I IFN response genes in 2250L (Fig. S2g). Differential gene expression analysis in 2250L treated with RT + ICI—which is the only condition where an abscopal response is observed—versus all other groups identified upregulation of interferon-stimulated genes and innate immune regulators (Fig. 2f–g). Among the most significantly induced genes was *Zbp1*, a necroptosis effector linking innate immune sensing to immunogenic cell death, which has previously been shown to be a critical mediator of DNA damage-induced tumor suppression and anti-tumor immunity (Fig. 2f–g, S2h)^37,38,45,46^. These findings implicate tumor-intrinsic responses to radiation—especially type I IFN signaling and necroptosis —as potential determinants of RT + ICI-induced abscopal responses.

### Immune Cell Features of the Abscopal Response

**Figure 3:**
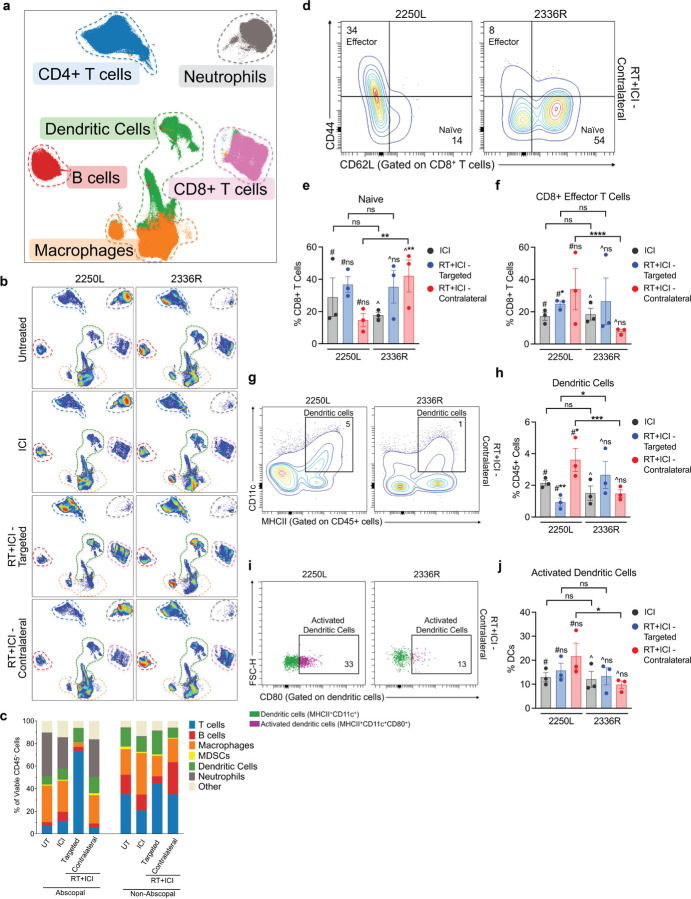
Enhanced CD8^+^ T effector cells and dendritic cell activation are associated with abscopal response. High parameter flow cytometry of abscopal 2250L and non-abscopal 2336R tumors from p53-null BALB/c mice harvested on day 10 of treatment and analyzed using OMIQ. (a) UMAP visualization of six major immune cell clusters in 2250L and 2336R tumors (files concatenated). Clusters manually annotated and outlined with dashed lines. (b) UMAP visualization of 2250L (left panel) and 2336R (right panel) tumors across untreated, ICI, RT+ICI–targeted, or RT+ICI–contralateral groups. Each UMAP concatenates three files per treatment. Colored dashed outlines denote annotated clusters shown in (a). (c) Percentage of viable CD45^+^ immune cell populations; n=3/group. (d) Representative (n=1/group) contour plots of naive (CD44^−^CD62L^+^) and effector (CD44^+^CD62L^−^) subsets gated from total CD8^+^ T cells in RT+ICI–contralateral tumors from 2250L and 2336R. Percentage of CD8^+^ T cells that are (e) naive or (f) effector in ICI, RT+ICI–targeted, or RT+ICI–contralateral tumors; n=3/group. (g) Representative (n=1/group) contour plots of dendritic cells (CD11c^+^MHCII^+^) gated from total CD45^+^ cells in RT+ICI–contralateral tumors from 2250L and 2336R. (h) Percentage of dendritic cells (CD11c^+^MHCII^+^) among total live CD45^+^ cells from 2250L and 2336R tumors after treatment; n=3/group. (i) Representative (n=1/group) contour plots of activated dendritic cells (CD80^+^) gated from total dendritic cells in RT+ICI–contralateral tumors from 2250L and 2336R (j) Percent activated dendritic cells (CD80^+^) of total dendritic cells from 2250L and 2336R tumors after treatment; n=3/group. (e-f, h, j) Immune cell subpopulations of interest plotted by tumor model (2250L and 2336R) and treatment (ICI in gray, RT+ICI–targeted in blue, and RT+ICI–contralateral in red). Tumor specific comparisons are denoted by # for 2250L and ^ for 2336R; comparisons between tumor models are shown in brackets. Mixed-effects models, * p<0.05, **p<0.01, ***p<0.001, ****p<0.0001.

To evaluate tumor immune microenvironment phenotypes in abscopal-competent versus non-abscopal tumor models, we optimized a 27-parameter stain set for high-dimensional spectral flow cytometry analysis on dissociated cells from tumors on day 10 after initiating therapy, capturing broad immune cell types of interest (Fig. 3a–c, Fig. S3)^47^.

Immunophenotyping revealed that the frequency of T cells increased in 2250L targeted tumors with RT + ICI, while the contralateral tumors and the non-abscopal 2336R model showed modest changes (Fig. 3c, S4c). In the contralateral tumors, naïve CD8^+^ T cells were significantly reduced, and effector CD8^+^ T cells were significantly increased after RT + ICI in the abscopal-competent 2250L relative to the non-abscopal 2336R (Fig. 3d–f). Moreover, contralateral tumors of the non-abscopal model had significantly reduced activated CD8^+^ T cells after RT + ICI compared with those of the abscopal model (Fig. S4d iii). Differences in CD4^+^ T and B lymphocytes between the two models were modest and variable (Fig S4e,h). Exploratory analyses of additional T-cell subsets—including memory, activation, and exhaustion states of CD4^+^ and CD8^+^ T cells, as well as gamma delta T cells and regulatory T cells—yielded variable results (Fig S4d-g). However, in the contralateral tumors of the non-abscopal model after RT + ICI, naïve CD4^+^ T cells were significantly increased with a significant corresponding reduction in effector CD4^+^ T cells compared to ICI, a pattern not observed in the abscopal model (Fig S4e ii-iv). In the contralateral tumors of the 2250L abscopal model, tissue resident memory CD4^+^ T cells were significantly increased after RT + ICI compared to ICI, whereas in the non-abscopal model, contralateral tumors showed a significant reduction in this subset (Fig S4e v). Total and activated (i.e., CD80+) dendritic cells were also significantly higher after RT + ICI in the contralateral tumor in the abscopal responsive 2250L compared to the non-abscopal 2336R (Fig. 3g–j). Together, these data indicate that abscopal responses to RT + ICI are characterized by tumor-intrinsic type I interferon signaling and necroptosis, local increases in macrophage phagocytic activity, and systemic induction of activated DCs and effector CD8^+^ T cells in distant, non-irradiated tumors.

### Enhancing Radiation-induced Type I IFN Responses through PARP7 Inhibition

**Figure 4:**
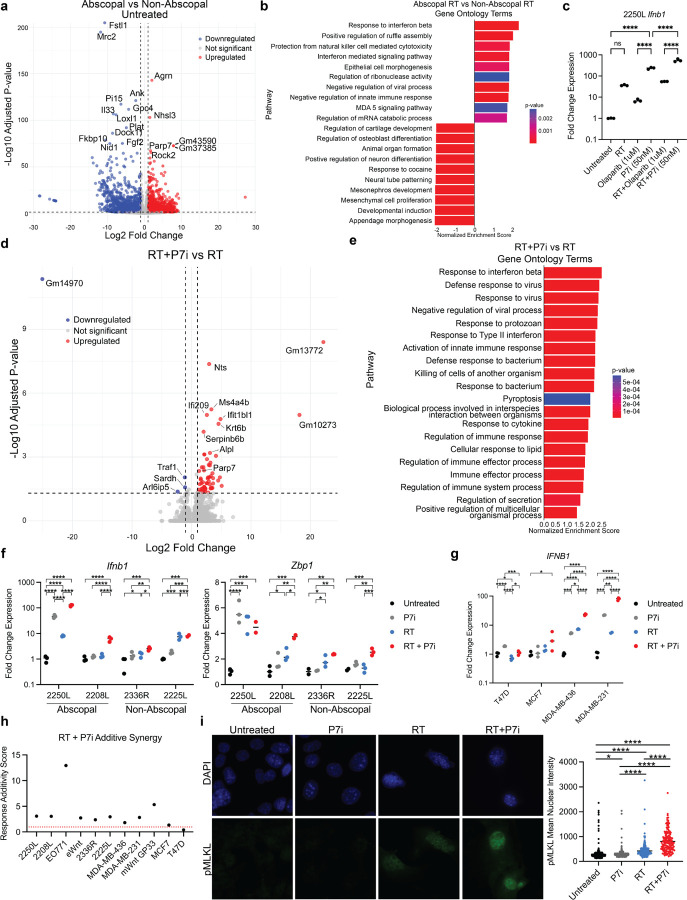
PARP7 inhibition synergizes with radiotherapy *in vitro* to enhance anti-tumor immune response. (a) Volcano plot of differential expressed genes (DEGs) from bulk RNA sequencing of untreated cultured tumor cells comparing abscopal-competent (2250L and 2208L) and non-abscopal-competent (2336R and 2225L) murine models. (b) Gene set enrichment analysis (GSEA) of irradiated abscopal-competent vs. non-abscopal-competent models using Gene Ontology (GO) terms. (c) Fold change in *Ifnb1* mRNA expression in 2250L murine cells 48 hours after treatments: untreated, RT, Olaparib (1μM), PARP7i inhibitor, RT+Olaparib (1μM), and RT+PARP7i (50nM). Fisher’s LSD test, * p<0.05, **p<0.01, ***p<0.001, ****p<0.0001. (d) Volcano plot of DEGs from bulk-RNA sequencing of abscopal-competent 2250L and 2208L murine cells treated with RT+PARP7i vs. RT alone. (e) GSEA comparing RT+PARP7i vs. RT in abscopal-competent murine samples using GO terms. (f) Fold change in *Ifnb1* (left) and *Zbp1*(right) mRNA expression across treatments (untreated, PARP7i, RT, RT+PARP7i) in abscopal-competent (2250L and 2208L) and non-abscopal-competent (2336R and 2225L) murine tumor cell lines. Fisher’s LSD test, * p<0.05, **p<0.01, ***p<0.001, ****p<0.0001. (g) Fold change in *IFNb1* mRNA expression across treatments (untreated, PARP7i, RT, RT+PARP7i) in human breast cancer lines (T47D, MCF7, MDA-MB-436, and MDA-MB-231). Fisher’s LSD test, * p<0.05, **p<0.01, ***p<0.001, ****p<0.0001. (h) RT+PARP7i additive synergy scores in murine and human breast cancer cell lines. (i) Immunofluorescence staining of 2250L cells by treatment. DAPI shown in blue and pMLKL in green. Mean nuclear pMLKL intensity (right) analyzed with Fisher’s LSD test, * p<0.05, **p<0.01, ***p<0.001, ****p<0.0001.

We next sought to identify tumor-intrinsic genetic modulators that could serve as therapeutic targets to potentiate the abscopal response. Bulk RNA sequencing of untreated tumor lines revealed differential baseline and RT-induced gene expression between abscopal-competent and non-abscopal models (Fig. 4a, S5a). GSEA of irradiated tumor lines demonstrated significant enrichment of interferon signaling and negative regulation of innate immune responses in abscopal models (Fig. 4b). Notably, one of the most enriched genes in abscopal models was *Parp7* (official gene name *Tiparp*), a mono-ADP-ribosyltransferase that suppresses type I IFN signaling and has been implicated as a therapeutic target in immunotherapy resistance (Fig S5b)^31,48,49^. PARP7 has previously been shown to suppress IFN signaling by MARylating and inactivating TBK1, a key kinase in the cGAS-STING-IRF3 axis^48^. We therefore hypothesized that inhibition of PARP7 would relieve this suppression and synergize with radiotherapy to enhance type 1 IFN responses.

Treatment with the selective PARP7 inhibitor RBN-2397 (PARP7i) induced an approximately 90-fold increase in *Ifnb1* expression in 2250L cells, markedly greater than the response observed with PARP1/2 inhibition using olaparib (Fig. 4c). Combination with radiation further amplified *Ifnb1* induction to ~ 500-fold, compared with only ~ 50-fold following RT + olaparib (Fig. 4c). Treatment with IAP (Inhibitor of Apoptosis Proteins) inhibitor xevinapant or ADAR1 inhibitor 8-Azaadenosine did not elicit increases in *Ifnb1* expression (Fig S5c). To assess global transcriptional effects, we performed bulk RNA sequencing in abscopal-competent models 2250L and 2208L (Fig. 4d). GSEA confirmed that PARP7i predominantly potentiated type I IFN pathway activation following RT (Fig. 4e).

RT-qPCR analyses further validated enhanced RT-induced interferon-stimulated gene (ISG) expression across both abscopal-competent and incompetent models (Fig. 4f, S5d-f). While PARP7i alone variably induced ISGs, the most consistent induction occurred with RT + PARP7i. In a panel of human breast cancer cell lines, PARP7i similarly showed greater-than-additive synergy with RT in driving *IFNB1* expression in most models tested (Fig. 4g–h), underscoring its broad potential as an amplifier of RT-induced ISG responses.

Because our earlier analyses implicated *Zbp1* in RT + ICI responses, we next examined the impact of PARP7i on necroptosis-related signaling. PARP7i increased RT-induced *Zbp1* expression in two of four models (Fig. 4f). In 2250L, *Zbp1* transcript levels were unchanged by PARP7i, but ZBP1 protein and phosphorylated MLKL (pMLKL)—a defining marker of necroptosis activation—were significantly increased (Fig. S5g). ZBP1 activation is triggered by Z-form nucleic acids (Z-NAs), which were induced by RT but not further augmented by PARP7i (Fig. S5h). By contrast, pMLKL accumulation was observed primarily in the RT + PARP7i condition, implicating PARP7i as an enhancer of RT-driven necroptosis engagement (Fig. 4i).

Together, these results identify PARP7 as a tumor-intrinsic brake on radiation-induced innate immune activation and demonstrate that PARP7 inhibition amplifies RT-driven type I IFN and necroptotic responses across diverse breast cancer models.

### PARP7 Inhibition Enhances the Abscopal Response In Vivo

**Figure 5:**
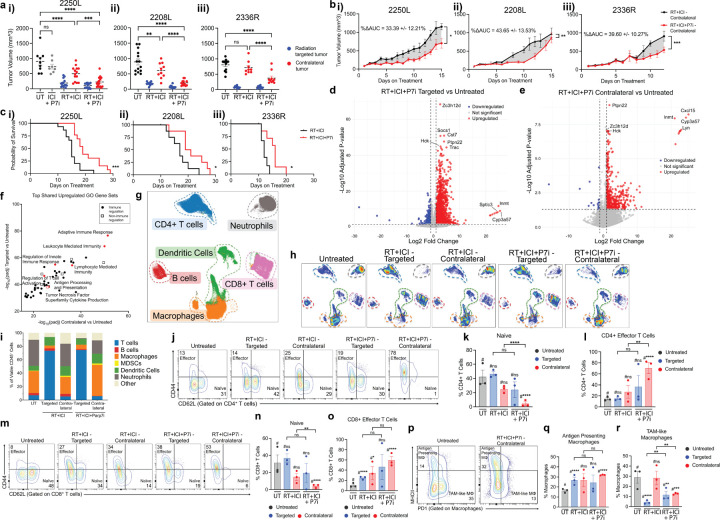
PARP7 inhibition augments the abscopal immune response to radioimmunotherapy. (a–c) *In vivo* response of abscopal-competent (2550L, 2208L) and non-abscopal (2336R) models to RT+ICI±PARP7 inhibition. (a) Day 10 tumor volumes comparing untreated (UT), ICI+PARP7i, RT+ICI, and RT+ICI+PARP7i in models (i) 2250L, (ii) 2208L, and (iii) 2336R. Fisher’s LSD test, * p<0.05, **p<0.01, ***p<0.001, ****p<0.0001. (b) ΔAUC of contralateral tumors in RT+ICI vs. RT+ICI+PARP7i; unpaired t-test, (i) p=0.0111, (ii) p=0.0024, (iii) p=0.0004. (c) Kaplan-Meier survival of RT+ICI and RT+ICI+PARP7i across models; (i) p=0.0006, (ii) p=0.0281, (iii) p=0.0104. (d-e) Volcano plots of differential expressed genes from bulk RNA-seq of abscopal-competent tumors (2250L and 2208L) comparing (d) RT+ICI+PARP7i–targeted vs untreated or (e) RT+ICI+PARP7i–contralateral vs untreated (n=3/treatment, day 10). (f) Top 50 shared upregulated gene ontology (GO) gene sets between RT+ICI+PARP7i–targeted vs untreated and RT+ICI+PARP7i–contralateral vs untreated in abscopal-competent tumors. Immune-related gene sets=black circles; non-immune gene sets=black squares; labeled gene sets=red. (g-r) High parameter flow cytometry of 2250L abscopal tumors on day 10 across treatments analyzed in OMIQ. (g) UMAP visualization of six major immune clusters across all treatments (files concatenated). (h) UMAPs across all treatment groups (n=3/group) with dashed outlines corresponding to clusters in (g). (i) Percentage of viable CD45^+^ immune populations; n=3/group. (j–o) Representative contour plots (n=1/group) and quantification (n=3/group) of naïve (CD44^−^CD62L^+^) and effector (CD44^+^CD62L^−^) subsets gated from (j-l) total CD4^+^ and (m-o) CD8^+^ T cells. (p-r) Representative contour plots (n=1/group) and quantification (n=3/group) of antigen-presenting macrophages (MHCII^+^PD1^−^) and tumor-associated macrophages (MHCII^−^PD1^+^) gated from total macrophages. (k-l, n-o, q-r) Immune cell subpopulations plotted by treatment grouped: untreated (gray), targeted (blue), and contralateral (red). Comparisons to untreated denoted by #; comparisons between treatments shown in brackets. Mixed-effects models, * p<0.05, **p<0.01, ***p<0.001, ****p<0.0001.

We next evaluated whether PARP7 inhibition augments abscopal effects *in vivo*. In the 2250L, 2208L, and 2336R models, mice were treated with RT + ICI with or without PARP7i. While PARP7i did not further suppress growth of irradiated tumors, its addition significantly reduced contralateral tumor volumes at the landmark timepoint relative to RT + ICI alone (Fig. 5a, S6a). Longitudinal analyses confirmed contralateral tumor growth inhibition over time (Fig. 5b, S6b-c) and improved survival (Fig. 5c), extending abscopal competence to 2336R, which was previously nonresponsive to radioimmunotherapy. Importantly, PARP7i + ICI, in the absence of RT, did not overcome ICI resistance (Fig. 5a i). Additionally, these enhancements of abscopal responses were specific to PARP7i and were not observed with the PARP1/2 inhibitor olaparib (Fig. S7).

RNA-seq of tumors (2250L, 2208L) from triple treated (RT + ICI + PARP7i) animals demonstrated overlapping transcriptional responses in irradiated and contralateral tumors, including upregulation of immune-related genes such as *Zc3h12d*, *Ptpn22*, and *Hck* relative to untreated control animals (Fig. 5d–e). GSEA of Gene Ontology (GO) gene sets revealed enrichment of innate immunity, antigen presentation, and adaptive immune response pathways, suggesting that triple therapy induces robust systemic immune activation (Fig. 5f).

To delineate the immunostimulatory effects of PARP7i at the cellular level, we performed spectral flow cytometry on dissociated tumors collected at day 10 (Fig. 5g–r). Targeted tumors showed broadly similar immune profiles between RT + ICI and RT + ICI + PARP7i (Fig. 5h–i). In contrast, contralateral tumors displayed distinct tumor immune microenvironment remodeling after PARP7i addition (Fig. 5i, S6d). Specifically, triple therapy reduced neutrophil infiltration while increasing macrophage abundance (Fig. 5i, S6l,n). Contralateral tumors exhibited increased effector CD4^+^ T cells and reduced naïve CD4^+^ T cells (Fig. 5j–l), with a parallel trend toward more effector CD8^+^ T cells (Fig. 5m–o). Exploratory analyses of T-cell subsets including memory, activation, and exhaustion states of CD4^+^ and CD8^+^ T cells, as well as gamma delta T cells—yielded variable results (Fig S6f-i). However, in the contralateral tumors of the triple therapy, both CD4^+^ T cells and CD8^+^ T cells showed more exhaustion and increased memory compared to RT + ICI alone (Fig S6f,h). Changes in B cells were less pronounced (Fig S6j).

Furthermore, PARP7i reduced the proportion of PD1^+^ immunosuppressive tumor-associated macrophages (TAMs) in the contralateral tumor while enriching for antigen presenting (MHCII^+^PD1^−^F4/80^+^) macrophages (Fig. 5p–r)^50,51^. Overall macrophage percentage increased in the contralateral tumors of the triple therapy, while changes in other myeloid cells were variable (Fig S6k-n).

Collectively, these data demonstrate that PARP7i enhances radioimmunotherapy-induced abscopal responses *in vivo*, and is associated with recruitment of antigen-presenting macrophages and effector CD4^+^ and CD8^+^ T cells in distant, non-irradiated tumors.

### ZBP1-Mediated Necroptosis is Required for the Abscopal Response

**Figure 6:**
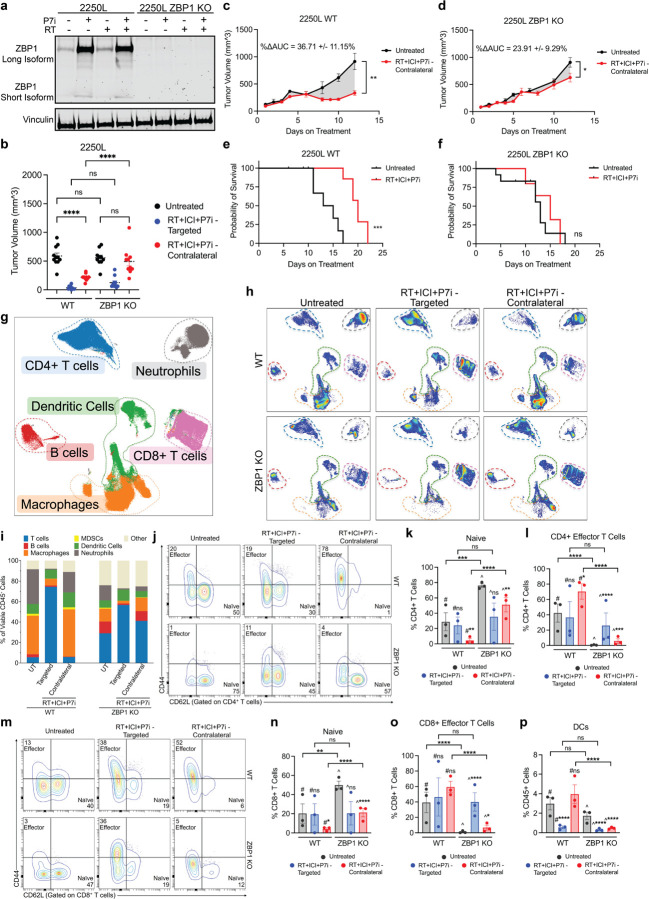
PARP7 inhibition enhances the abscopal response through ZBP1-dependent necroptosis. (a–f) *In vivo* effects of PARP7 inhibition in 2250L wild-type (WT) and ZBP1 knockout (KO) tumors. (a) Western blot of long and short ZBP1 isoforms in 2250L WT and ZBP1 KO cells treated with PARP7i and/or radiation (RT). Vinculin loading control. (b) Static tumor volume on day 10 of treatment across groups (untreated, RT+ICI+PARP7i–targeted and RT+ICI+PARP7i–contralateral). Fisher’s LSD test, *p<0.05, **p<0.01, ***p<0.001, ****p<0.0001. (c-d) ΔAUC of untreated vs RT+ICI+PARP7i contralateral tumor; unpaired ttest, (c) 2250L WT, p=0.0021, (d) 2250L ZBP1 KO, p=0.0138. Comparisons based on the time at which half of the control group reached tumor burden. (e-f) Kaplan-Meier survival analysis comparing untreated and RT+ICI+PARP7i groups; (e) 2250L WT, p=0.0004, (f) 2250L ZBP1 KO, p=0.5383. (g-p) High parameter flow cytometry of 2250L WT and ZBP1 KO tumors on day 10 across untreated, RT+ICI+PARP7i–targeted and RT+ICI+PARP7i–contralateral groups, analyzed in OMIQ. (g) UMAP visualization of six major immune clusters in WT and ZBP1KO tumors (files concatenated). Clusters manually annotated and outlined with dashed lines. (h) UMAP visualizations of WT (top panel) and ZBP1 KO (bottom panel) tumors (n=3 per map; files concatenated). Dashed colored outlines denote annotated clusters shown in (g). (i) Percentage of viable CD45^+^ immune populations; n=3/group. (j-o) Representative contour plots (n=1/group) and quantification (n=3/group) of naive (CD44^−^CD62L^+^) and effector (CD44^+^CD62L^−^) subsets gated from total (j-l) CD4^+^ T cells and (m-o) CD8^+^ T cells. (p) Percentage of dendritic cells (CD11c^+^MHCII^+^) among total live CD45^+^ cells (n=3/group). (k-l, n-p) Immune cell populations plotted by treatment: untreated (gray), RT+ICI+PARP7i–targeted (blue), and RT+ICI+PARP7i–contralateral (red). Comparisons to WT untreated tumors denoted by #; comparisons to ZBP1 KO untreated tumor by ^. Comparisons between tumor models are shown in brackets. Mixed-effects models, * p<0.05, **p<0.01, ***p<0.001, ****p<0.0001.

Because PARP7i enhanced RT-induced necroptosis *in vitro*, we next tested the requirement for tumor-intrinsic ZBP1 *in vivo*. Using CRISPR-mediated knockout in 2250L cells, we disrupted both long and short ZBP1 isoforms, abolishing its upregulation by RT + PARP7i (Fig. 6a).

In bilateral tumor models, ZBP1 deficiency abrogated the enhanced abscopal response observed in WT tumors treated with RT + ICI + PARP7i, as measured by contralateral tumor growth (Fig. 6b–d, S8a-b) and survival (Fig. 6e–f). Growth of irradiated or untreated tumors was not significantly affected by ZBP1 deficiency (Fig. S8a-b). Thus, tumor-intrinsic necroptosis is selectively required for PARP7i-mediated enhancement of abscopal responses to radioimmunotherapy.

Spectral flow cytometry of WT and ZBP1-KO tumors on day 10 revealed that the most striking treatment-dependent differences were in contralateral T cell populations (Fig. 6g–i, Fig S8c-d). In ZBP1-deficient tumors, naïve CD4^+^ and CD8^+^ T cells persisted in contralateral, unirradiated tumors, with a significant reduction in effector CD4^+^ and CD8^+^ T cell subsets after RT + ICI + PARP7i compared to WT (Fig. 6j–o). ZBP1-deficient contralateral tumors showed decreased memory CD4^+^ and CD8 + T cell subsets (Fig S8e-f). In addition, contralateral tumors also exhibited reduced dendritic cell abundance following triple therapy in the absence of ZBP1 (Fig. 6p). ZBP1-KO tumors demonstrated variable changes in B cell and myeloid populations in both the targeted and contralateral tumors (Fig. S8g-k).

In summary, tumor-intrinsic ZBP1-dependent necroptosis is necessary for recruitment of APCs and effector CD4^+^ and CD8 + T cells in distant, non-irradiated tumors, and is essential for abscopal responses to RT + ICI + PARP7i.

### Human Breast Cancer PARP7 Signature Correlates With Known Inflammatory Signatures

**Figure 7:**
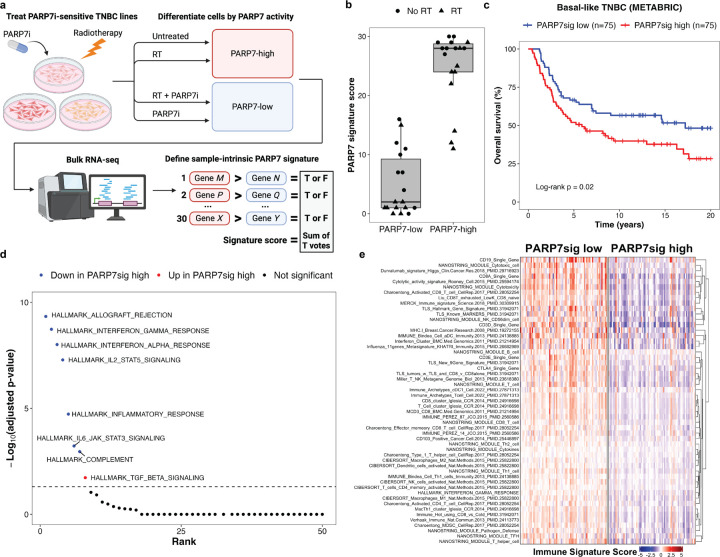
Sample intrinsic PARP7 gene signature correlates with known inflammatory signatures in basal-like TNBC. (a) A sample-intrinsic PARP7 gene signature was derived from *in vitro*treatment (untreated, RT, PARP7i, or RT+PARP7i) of PARP7i-responsive murine TNBC cell lines (2250L, 2208L, and mWnt) using the k-Top Scoring Pairs approach (n=3 per cell line per treatment). (b) Application of the PARP7 signature distinguished the *in vitro* treatment groups. Circles denote no RT and triangles denotes RT-treated samples. (c) Kaplan-Meier survival analysis of patients with primary basal-like TNBC, stratified by PARP7 signature score using pretreatment microarray data from the METABRIC study (n=150). (d) Comparison of PARP7 signature groups in the METABRIC basal-like TNBC dataset using Hallmark gene sets. Signature scores for each sample were calculated as the median expression of each gene set and were compared using Mann-Whitney U-tests with post-hoc Bonferroni correction. For significant gene sets (adjusted p<0.05), color denotes directionality (blue = down in PARP7sig high group, red = up in PARP7sig high group, black = not significant). (e) Comparison of PARP7 signature groups using a curated set of immune signatures in the METABRIC basal-like TNBC dataset. Of 124 immune signatures tested, 53 were significantly different between groups (Mann-Whitney U-tests with Bonferroni correction and conservative threshold of adjusted p<0.0001).

Given the role of PARP7 as a negative regulator of the anti-tumor immune response *in vivo*, we hypothesized that PARP7 may perform similar functions in patients with TNBC. We utilized the k-Top Scoring Pairs (k-TSP) approach to generate a 30-TSP PARP7 gene signature derived from three PARP7i-sensitive murine TNBC cell lines treated with PARP7i and/or RT (Fig. 7a)^52^. This approach assesses the sample-intrinsic non-parametric relationships between gene pairs to generate a signature score, thereby facilitating robustness to differences in experimental approach across platforms used for transcriptome profiling^53^. The 30-TSP PARP7 gene signature contained gene pairs with multiple ISGs including *TRIM34*, a positive regulator of ZBP1^54^, as well as *TIPARP*, which encodes PARP7 (Fig. S9a). Application of this signature to the *in vitro* samples validated its ability to distinguish samples by PARP7i treatment (Fig. 7b).

In patients with basal-like TNBC in the METABRIC cohort, high PARP7 signature expression using a median score cutoff was correlated with worse survival (Fig. 7c). To evaluate whether the PARP7 signature is anticorrelated with markers of immune activation, we compared the expression of Hallmark gene sets between patients stratified by PARP7 signature score. While less than 20% of the Hallmark gene sets are immune-related, PARP7 signature groups were significantly differentiated by only immune gene sets, with high PARP7 signature score anticorrelating with pro-inflammatory gene sets and positively correlating with the immunosuppressive tumor growth factor beta (TGF-β) signaling gene set (Fig. 7d). To further examine this relationship, we compared PARP7 signature groups using a curated set of 124 immune gene signatures previously used in the analysis of human TNBC^55^, and all 53 signatures that were significantly different between groups were decreased in the high PARP7 signature group, including signatures for cytotoxic T cells and tertiary lymphoid structures (Fig. 7e). Relationships between the PARP7 signature and known inflammatory signatures were recapitulated in the TCGA basal-like TNBC cohort (Fig. S9b-d), as well as in HR + breast cancer in the METABRIC cohort (Fig. S9e), albeit without the difference in survival in the TCGA cohort likely due to its known limitations for survival analysis^56^. Taken together, these findings suggest that PARP7 acts as a negative regulator of the anti-tumor immune response in human breast cancer.

## DISCUSSION

Our study addresses the heterogeneity of abscopal responses to radioimmunotherapy in breast cancer and identifies tumor-intrinsic mechanisms that govern these outcomes. Using a panel of clinically representative p53-null murine mammary models that capture both molecular and tumor immune microenvironment diversity in immunotherapy-resistant TNBC, we found that only a subset demonstrated reproducible abscopal effects after RT + ICI. Abscopal-competent tumors were distinguished by enhanced type I IFN signaling, ZBP1 induction, and macrophage phagocytic activity at the irradiated site, coupled with systemic recruitment to distal tumor sites of antigen-presenting cells and effector T cell populations. These findings highlight the importance of tumor intrinsic innate immune signaling, in addition to adaptive immune responsiveness, in determining whether local radiation during radioimmunotherapy translates into systemic immunity.

**Figure 8:**
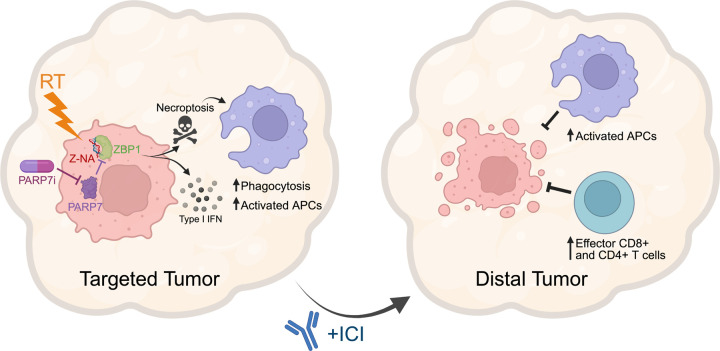
Schema of PARP7 inhibition enhancing necroptosis-dependent abscopal immunity to radioimmunotherapy. Inhibition of PARP7 in the radiotherapy (RT)–targeted tumor enhances ZBP1 activation, which is triggered by RT-induced Z-form nucleic acids (Z-NAs). This activation promotes necroptosis and type I interferon (IFN) production, leading to increased abundance, phagocytic activity, and activation of professional antigen presenting cells (APCs). In the distant, non-irradiated tumor, these effects drive enhanced activation of APCs and increased infiltration of effector CD4^+^ and CD8^+^ T cells compared to radioimmunotherapy alone.

A central discovery from our work is the identification of PARP7 as a tumor-intrinsic suppressor of radiation-induced innate immune signaling. PARP7 inhibition amplified RT-driven type I IFN responses and promoted ZBP1-dependent necroptosis (Fig. 8). Notably, this synergy was not reproduced by PARP1/2 inhibition, underscoring that the immunomodulatory effects of PARP7 are distinct from DNA repair-focused PARP family members. These findings build on prior evidence that PARP7 suppresses TBK1 and IFN signaling^31,57^, extending its relevance to radiation-associated innate immune responses.

Mechanistically, tumor-intrinsic necroptosis was required for the PARP7i-mediated systemic anti-tumor immunity. ZBP1 deficiency abrogated antigen-presenting cell recruitment and prevented conversion of naïve CD4^+^ and CD8 + T cells into antigen-exposed effector populations in distant, non-irradiated tumors (Fig. 8). ZBP1 may also stimulate radiation-induced tumor cell phagocytosis, as a recent study has shown that inhibiting the phagocytosis inhibitor CD47 resulted in expansion of APCs and effector T cell responses in distant, non-irradiated tumors to potentiate abscopal responses^58^. How local RT-induced necroptosis induces systemic immune alterations remains to be clarified but may involve immunostimulatory extracellular vesicles generated during RT-induced necroptotic cell death that traffic to distant tumor sites^59–61^. This raises the possibility that necroptotic signaling not only shapes the local tumor microenvironment but also licenses distant tumors for radioimmunotherapy response. Together, these results advance our understanding of the determinants of abscopal responses and nominate PARP7 inhibition, in combination with RT + ICI, as a promising therapeutic strategy in ICI-resistant breast cancers. More broadly, they highlight tumor-intrinsic innate immune signaling and necroptosis engagement as both biomarkers and mechanistic underpinnings of systemic radioimmunotherapy responses.

Clinically, PARP7 inhibitors are already being tested with ICI as a strategy to overcome ICI resistance in breast and other cancer types (NCT04053673, NCT05127590). Our data refine this paradigm by showing that PARP7i alone, or with ICI, may not be sufficient in some tumors; however, synergy with concurrent radiotherapy can uncover systemic anti-tumor immunity. Indeed, RT + ICI + PARP7i combination treatment elicited abscopal responses even in models previously resistant to radioimmunotherapy. These results highlight radiation as a synergistic therapeutic partner with PARP7i and ICI that should be investigated in future clinical trials.

Recent studies have highlighted prognostic features that influence radioimmunotherapy response heterogeneity in non-small cell lung cancer^11,20,22^, melanoma^62^, and TNBC^63^, underscoring the importance of biomarkers to guide therapy in ICI-resistant tumors. The PARP7 gene signature developed in this study correlates with poor prognosis and immune suppression in TNBC patients, and may serve as a predictive biomarker for future clinical trials evaluating PARP7i with concurrent radioimmunotherapy in ICI-resistant cancers.

## Materials and Methods

### Genetically engineered mouse models

All animal work was approved by the UNC Institutional Animal Care and Use Committee (IACUC). BALB/c mice were obtained from The Jackson Laboratory (JAX stock #000651). Blinding of animal experiments was not performed.

#### Tumor models and implantation

Tumor lines 2250L, 2208L, 2336R, and 2225L were obtained as previously described^42,43^ and transplanted into BALB/c mice for expansion. Tumors dissociated using the Miltenyi Biotec Tumor Dissociation Kit (Miltenyi Biotec, 130-096-730) according to the manufacturer’s instructions. Mice received bilateral fat pad injections into the fourth mammary gland containing 5 × 10^5^ tumor cells suspended in a 1:1 mixture of HBSS and Cultrex UltiMatrix Reduced Growth Factor Basement Membrane Extract (Bio-Techne, BME001–10).

#### Monitoring, randomization, and treatment start

Mice were palpated three times per week for mammary tumor development. Once a tumor reached 6 mm in its longest dimension (defined as day 1 of treatment), mice were randomized to the indicated treatment groups and were subsequently monitored three times per week for the remainder of the study.

#### Drug treatments

Following randomization, mice assigned to dual immune checkpoint inhibition received anti-PD-1 (BioXCell, BE0146; 10 mg/kg) and anti-CTLA-4 (BioXCell, BE0164; 5 mg/kg) diluted in sterile PBS and administered intraperitoneally twice weekly. Mice assigned to PARP7 inhibition received PARP7 inhibitor, RBN-2397 (MedChemExpress, HY-136174; 30 mg/kg), diluted in sterile PBS and administered by oral gavage once daily for the duration of treatment. Mice assigned to olaparib received olaparib (MedChemExpress, HY-10162; 50 mg/kg) diluted in PBS via oral gavage twice daily. In select cohorts, Olaparib was provided as formulated chow (Research Diets Inc.; 625 mg/kg).

#### Irradiation

Mice assigned to radiotherapy received irradiation using the SmART Image-guided irradiator, targeting a single tumor with 8Gy x 3 fractions (three beams). Radiation was delivered every 24 hours for a total of 3 days (treatment days 1–3).

#### Tumor measurements and endpoints

Tumor length and width were measured with calipers, and volume was calculated as d^2^ x D/2 (d=shortest diameter; D=longest diameter). Mice were euthanized in a humane manner in accordance with the guidelines set by the UNC IACUC at a predefined experimental endpoint. Mammary tumors were collected at necropsy and divided into three pieces for downstream analyses.

#### Housing conditions for mice

At the Division of Comparative Medicine, the standard light cycle for mice is set from 07:00 to 19:00. The ambient temperature is maintained between 20 and 23 °C, with a humidity level ranging from 30% to 70%. A maximum of five adult mice of the same sex were housed per cage.

### Spatial Transcriptomics

Spatial transcriptomics was conducted using the NanoString GeoMx Digital Spatial Profiler (DSP) with the murine Whole Transcriptome Assay. This platform profiles regions of interest (ROIs) within formalin-fixed paraffin-embedded (FFPE) tissues; sequencing of released oligos was performed downstream. On day 10 of treatment, n=3 mice per treatment group were euthanized in a humane manner in accordance with UNC IACUC guidelines. Tissues were fixed in 10% neutral buffered formalin (NBF) for 24 h, then submitted to the UNC Pathology Services Core for paraffin embedding and downstream processing.

From each tumor, two cores were randomly selected from FFPE tissue and arranged into a tissue microarray, totaling six cores per treatment group.

The FFPE microarray was sectioned at 5 μm onto positively charged slides (Nanostring, 100473). Sections were deparaffinized in CitriSolv (3 changes, 5 min each), then rehydrated with 100% EtOH (2 changes, 5 min each) followed by 95% EtOH (1 change, 5 min). Slides were dipped into DEPC-treated water and transferred into 1x Tris-EDTA (99°C for 20 min). After incubation, slides were moved to room temperature 1x PBS for 5 min. To expose RNA targets, slides were incubated in Proteinase K (0.1 μg/mL) solution for 15 min. Slides were then washed in 1x PBS, post-fixed in 10% NBF for 5 min, washed twice in NBF Stop Buffer (5 min each), and rinsed in 1x PBS. Post-fixation served to preserve soft-tissue morphology.

In situ hybridization solution was prepared according to manufacturer’s specifications with Buffer R (Nanostring, 121300313), Whole Transcriptome Atlas Probe Mix (Nanostring, 121401103), and DEPC-treated water. The hybridization solution was added to the slide, and a Grace Bio-Labs HybriSlip was applied. The slide was incubated at 37°C overnight. Following incubation, to remove unbound/off-target probes, the slide was washed with 2x SSC, followed by two washes in Stringent Wash Solution (1:1 4x SSC:formamide) at 37°C for 25 min each, then 2 washes in 2x SSC for 2 min each.

The tissue was then stained for PanCK (Nanostring, 220507–01) and CD45 (Nanostring, 210708–01). The slide was blocked with Buffer W (121300313) for 30 min, protected from light. The morphology marker solution was prepared with PanCK, CD45, and SYTO 13 nuclear stain (Nanostring, 121300303). The slide was stained for 1 h, then washed twice in 2x SSC for 5 min each. Following staining, the slide was loaded onto the GeoMx DSP.

ROIs were drawn on digital slide images such that each area of interest (PanCK^+^ or CD45^+^) contained a minimum of 100 nuclei when possible and were selected near, but excluding, necrotic regions when possible. Two cores from one tumor were excluded from ROI selection due to insufficient nuclei. Oligo-labeled probes were hybridized to slides, which were then cleaved by UV exposure at the targeted ROIs. Released oligos were pooled and sequenced on a NextSeq2000 P2 with paired-end sequencing at 2×27 base pairs according to NanoString GeoMx specifications.

#### Data analysis

Whole transcriptome data were uploaded into R studio using a combination of NanoString-developed (GeoMxTools & NanoStringNCTools) and open-source R packages. Quality control (QC) and normalization were conducted in R. Adequate tissue sampling was assessed using recommended thresholds for number of raw sequencing reads, percent sequencing saturation, negative probe count, and no template control count. One ROI was removed due to poor expression. Normalization was conducted using upper quartile (Q3) scaling to the 75^th^ percentile, accounting for variability in the data. Differential expression analysis was performed using DESeq2. Gene Set Enrichment Analysis (GSEA) was conducted using the Mouse Molecular Signatures Database using the Gene Ontology gene sets^64,65^.

### Tumor percentage quantification on H&E slides

All tissues used for H&E staining were formalin-fixed in 10% NBF for 12 hours and subsequently transferred to 70% ethanol. Fixed tissue was sent to HistoWiz (Brooklyn, NY, USA) for paraffin – embedding, hematoxylin and eosin (H&E) staining, and digitally scanning to generate high-resolution H&E & whole-slide images. The tumor percentage on H&E-stained slides was quantified using QuPath (version 0.5.1), an open-source software for digital pathology image analysis^66^. Briefly, a pixel-based thresholder was applied to define the total analyzed tissue area, and fragmented tissue or artifacts were manually excluded. For classifier training, a composite training image was created using a 10,000 μm² square region from each representative sample, including both epithelial tumor and stromal components. Within the training image, epithelial tumor areas were annotated against stroma, necrosis, hemorrhage, and fibrosis. A built-in pixel classifier in QuPath was trained on these annotations to generate an epithelial tumor vs. other classifier, which was subsequently applied to all slides. All slides underwent blinded manual review to correct misclassified regions. Tumor percentage was calculated as the ratio of epithelial tumor area to the total analyzed tissue area.

### Spectral flow cytometry

On day 10 of treatment, n=3 mice per treatment group were euthanized by CO_2_ euthanasia in accordance with UNC IACUC guidelines. Tumor digestion and flow cytometry staining were performed as described in Green et al.^47^. Briefly, tumors were minced and enzymatically digested for 30 min at 37°C in RPMI-1640 supplemented with 5% bovine growth serum, 1.25 mg/mL Collagenase IV (Worthington), 1 mg/mL DNase I (Worthington), and 0.1% soybean trypsin inhibitor (Worthington). Digested cell suspensions were filtered through 70 μm strainers (Fisher) and centrifuged at 1600 rpm for 5 min. Pelleted cells were then washed with FACS buffer (2% bovine growth serum in DPBS) and stained for flow cytometry analysis.

The 27-parameter stain set for high-dimensional spectral flow cytometry, including each marker, clone, fluorochrome, dilution, supplier, catalog number, and antibody concentration, is provided in Supplementary Table 1. Cell suspensions were first incubated with Fc receptor blocker (1:500, BioLegend) and stained with LIVE/DEAD Fixable Blue (1:800, Invitrogen) in FACS buffer for 10 min at room temperature in the dark. Fluorescent antibody master mixes were prepared in BD Horizon Brilliant Stain Buffer (BD Biosciences). Cells were incubated with surface antibodies for 30 min at 4°C in the dark. Cells were washed with FACS buffer and fixed with 1x FoxP3 Fix/Perm buffer for 30 min at 4°C, followed by intracellular transcription factor staining according to the manufacturer’s protocol (Foxp3/Transcription Factor Staining Buffer Set, Invitrogen).

All events were acquired on a Cytek Aurora 5-laser spectral flow cytometer, unmixed in SpectroFlo using single-stained compensation controls, and analyzed using OMIQ software from Dotmatics (www.omiq.ai). Uniform manual gating was performed to exclude debris and doublets, with live CD45^+^ immune cells used for downstream analysis (representative gating strategy in Fig. S3). For dimension reduction, all samples were subsampled to an equal number of live CD45^+^ cells and jointly analyzed in OMIQ using UMAP with uniform settings (neighbors = 30, correlation metric, minimum distance = 0.4) across datasets to enable direct comparison. Cell populations of interest identified in OMIQ were expressed as a percentage of a parent population and compared across groups using mixed-effects logistic regression models with a fixed effect for treatment group and a random intercept for each tumor. Volcano plots were generated using ggplot2, and statistical models were built using the glmmTMB package in RStudio V2023.06.1 running R V4.4.2 (R Core Team, Vienna, Austria). Bar graphs were made using Prism 10 version 10.5.0.

### Establishment of stable tumor cell lines

#### Generation of tumor cell lines

Tumors were dissociated using the Miltenyi Biotec Tumor Dissociation Kit (Miltenyi Biotec, 130-096-730) according to the manufacturer’s instructions. The dissociated tumor cells were cultured in HuMEC Ready Medium (Thermo Fisher Scientific, 12752010) supplemented with 5% FBS (VWR, 76294–120) and selected with Geneticin (G418) (Thermo Fisher Scientific, 10131035; 200 μg/mL), leveraging using the neomycin resistance conferred by the p53-null allele. Once the stable cell lines were established, they were validated using bulk RNA sequencing (RNA-seq) to confirm that each line was genetically and phenotypically matched to its parent tumor.

Cells were maintained in MammaryLife^™^ Basal Medium (Lifeline Cell Technology, LM-0041) supplemented with 10% FBS (VWR, 76294–120). All cells were cultured at 37 °C in a humidified incubator with 5% CO_2_ in the air and were tested monthly for mycoplasma using MycoStrip^®^ Mycoplasma Detection Kit (Invivogen, rep-mysnc-100).

#### Generation of ZBP1 KO tumor line

Neon transfection (Invitrogen, MPK1025) was performed using recombinant Cas9 purified by the UNC Center for Structural Biology Protein Expression and Purification Core), crRNA (Integrated DNA Technologies (IDT), guide #1: 5’- GGCGGUAAAGGACUUGAUUG-3’; guide #2: 5’-CCCUGUGAAGAUUGGCCAGC-3’; guide #3: 5’- GGGUUCCUCCCCAGACAAUC-3’) and tracrRNA (IDT, 1072532) using the manufacturer’s protocol and recommended electroporation settings. Western blot and Sanger sequencing confirmed gene targeting and functional assays (Amplification primers: Forward: 5’- GGGTCTGCTCTGCCTACATG-3’; Reverse: 5’-TTACCATTTGGCCACCCAGA-3’. Sequencing primer: 5’-GTCTGCTCTGCCTACATGACAGATTAC-3’). The following antibody was used for pathway readouts: anti-MLKL phosphor S345 (Abcam, ab196436; 1:500).

### Immunofluorescence

Cells were plated at 50,000 cells/well in 6-well plates. 24 h after seeding, cells were treated with DMSO, PARP7i (50nM), DMSO+8Gy RT, and PARP7i (50nM)+8Gy RT. 72 h after treatment, cells were fixed in 4% formaldehyde for 15 min at room temperature. Cells were permeabilized in 0.2% Triton X-100 in PBS for 10 min. Fixed and permeabilized cells were incubated in blocking solution (1% BSA in PBS) for 30 min at room temperature followed by incubation with primary antibodies (in 1% BSA in PBS) at 4 °C overnight. After incubation in primary antibody, cells were washed 3x with PBS and incubated with DAPI for DNA counterstain and Alexa Fluor-conjugated anti-mouse or anti-rabbit secondary antibodies. Cells were washed 3x with PBS and mounted on slides using ProLong^™^ Gold Antifade Mountant (Invitrogen, P36934). Fluorescence images were acquired on an Olympus BX61 upright fluorescence microscope. Image analysis was performed using ImageJ (Fiji).

The following primary antibodies were used for immunofluorescence: anti-MLKL phosphor S345 (Abcam, ab196436; 1:500) and anti-ZBP1 mAb (Zippy-1) (AdipoGenLife Sciences, AG-20B-0010-C100; 1:500). The following secondary antibodies were used: Goat anti-Rabbit IgG (H+L) Cross-Adsorbed Secondary Antibody, Alexa Fluor^™^568 (Thermo Fisher Scientific, A-11011; 1:500) and Goat anti-Mouse IgG (H+L) Cross-Adsorbed Secondary Antibody, Alexa Fluor^™^ 488 (Thermo Fisher Scientific, A-11001;1:500).

### Quantitative PCR with reverse transcription (RT–qPCR)

Cells were plated at 150,000 cells/well in 6-well plate. 24 h after seeding, cells were treated with DMSO, PARP7i (50nM), DMSO+8Gy RT, and PARP7i (50nM)+8Gy RT. 48 h after treatment, RNA was isolated using RNeasy Plus Mini Kit (Qiagen, 74136) according to manufacturer’s instructions. RNA concentrations were measured with the Qubit RNA Broad Range kit (Thermo Fisher Scientific, Q10210). cDNA was synthesized with the iScript cDNA Synthesis kit (BioRad, 1708891) using 1 μg RNA. RT–qPCR was performed using Fast SYBR Green master mix (Thermo Fisher Scientific, 4385617) on a QuantStudio 6 Real-Time PCR system (Thermo Fisher Scientific). Cycling conditions were 95 °C for 15 min, followed by 40 two-step cycles (95 °C for 15 s; 60 °C for 60 s).

The following primer sequences were used: mIFNb Forward: 5’ – GAGCTCCAAGAAAGGACGAAC – 3’, mIFNb Reverse: 5’ – GGGAGTGTAACTCTTCTGCAT – 3’, mIFNa Forward: 5’ GGATGTGACCTTCCTCAGACTC – 3’, mIFNa Reverse: 5’ – ACCTTCTCCTGCGGGAATCCAA – 3’, mCXCL10 Forward: 5’ – GGTGAGAAGAGATGTCTGAATCC – 3’, mCXCL10 Reverse: 5’ – GTCCATCCTTGGAAGCACTGCA – 3’, mCCL5 Forward: 5’ – CCTGCTGCTTTGCCTACCTCTC – 3’, mCCL5 Reverse: 5’ – ACACACTTGGCGGTTCCTTCGA – 3’, mIFIT1 Forward: 5’ – TACAGGCTGGAGTGTGCTGAGA – 3’, mIFIT1 Reverse: 5’ – CTCCACTTTCAGAGCCTTCGCA – 3’, mZBP1 Forward: 5’ – GGGTCTGCTCTGCCTACATG – 3’, mZBP1 Reverse: 5’ – TTACCATTTGGCCACCCAGA – 3’, hIFNB1 Forward: 5” - CTTGGATTCCTACAAAGAAGCAGC – 3’, hIFNB1 Reverse: 5’ - TCCTCCTTCTGGAACTGCTGCA – 3’.

Additive synergy score was calculated as the ΔΔC_t_ of *Ifnb1* or *IFNB1* induced compared to DMSO of the combinatory treatment, RT+PARP7i, divided by the sum of both treatments administered separately.

### Bulk-RNA Sequencing

mRNAseq data of treatment naïve mouse mammary tumors from Hollern et al. (GEO: GSE124821)^44^ and Sutcliffe et al. (GEO: GSE223630)^67^ were utilized in this study.

Cells were plated at 50,000 cells/well in 6-well plate. 24 h after seeding, cells were treated with DMSO, PARP7i (50nM), DMSO+8Gy RT, and PARP7i (50nM)+8Gy RT. 48 h after treatment, RNA was collected using Qiagen RNeasy kit. Samples were sent to the Translational Genomics Lab at UNC for sequencing. RNA quality and integrity were assessed using the TapeStation, and samples with RNA Integrity Number (RIN) > 5 were used for library preparation.

RNA-seq libraries were prepared using Illumina TruSeq Stranded mRNA Library Prep Kit, and sequencing was performed on an Illumina NextSeq 2000 platform. Libraries were sequenced using paired-end 2×50 bp reads (100-cycle kit, P3 flow cell). Sequencing was performed with a single pool of up to 30 libraries across one lane, with a target output of 120 Gb. Each library was sequenced to a depth of approximately 50 million clusters (i.e., paired-end fragments).

### Bioinformatics Processing and Quality Control

Raw sequencing data (FASTQ files) were processed using a standardized RNA-seq pipeline. Where applicable, multiple FASTQ files per sample were merged prior to processing. Adapter trimming and removal of low-quality bases were performed using fastp (v0.24.0). Quality control (QC) of raw and trimmed reads was conducted using FastQC (v0.12.1), and summary statistics before and after trimming were compiled from fastp output.

Reads were aligned to the Mus musculus GRCm39 using STAR aligner (v2.7.11b). Gene expression quantification was performed using Salmon (v1.10.3) in alignment-based mode, generating both transcripts per million (TPM) values and raw counts for downstream analysis.

Normalized gene expression values were computed using upper quartile normalization, in which the 75th percentile expression value for each sample was scaled to 1000. This normalization was performed using a custom R script (quantile75to1k.R), producing “quantile75eq1k” values for comparison across samples.

Alignment metrics were evaluated using Picard (v2.22.4, CollectRnaSeqMetrics) and samtools (v1.10, flagstat) to calculate read alignment statistics including total reads, mapped/unmapped reads, and maximum read length. QC metrics across all samples were aggregated using MultiQC (v1.27.1). A comprehensive quality control summary was generated in an HTML report (RNA_QC_report.html). Expression matrices were merged across all samples for subsequent analysis.

Differential expression analysis was performed using DESeq2. Gene Set Enrichment Analysis (GSEA) was conducted using the Mouse Molecular Signatures Database using the Gene Ontology gene sets^64,65^.

### Generation of the PARP7 gene signature

Bulk RNA-seq transcriptomes from the 2250L, 2208L, and mWnt murine cell lines treated in triplicate with DMSO, PARP7i (50nM), DMSO+8Gy RT, or PARP7i (50nM)+8Gy RT were grouped as “PARP7-high” (DMSO or RT alone) or “PARP7-low” (PARP7i or RT+PARP7i). Murine transcriptomes with TPM normalization were mapped to their human orthologs using the HMD_HumanPhenotype.rpt file obtained from the Mouse Genome Informatics database, and these humanized transcriptomes were filtered to contain genes that were present in the human breast cancer METABRIC Illumina HT-12 v3 microarray dataset. The PARP7 gene signature was generated using the k-Top Scoring Pairs (k-TSP) approach implemented in the switchBox R package^52^. A 30-TSP signature was generated to distinguish the PARP7-high and PARP7-low *in vitro* groups, with gene pairs selected from the top 800 differentially expressed genes between the DMSO and RT+PARP7i treatments using the SWAP.Filter.Wilcoxon filter function. Scoring of the 30-TSP PARP7 signature was performed using the SWAP.KTSP.Statistics function.

The 30-TSP PARP7 gene signature was applied to the breast cancer METABRIC Illumina HT-12 v3 microarray dataset and the RSEM batch-normalized TCGA breast cancer dataset obtained from cBioPortal. For scoring Hallmark and curated immune gene sets^55^, gene sets for which at least 95% of genes were present in the human dataset were scored as median expression. Gene signature scores were compared using Mann-Whitney U-tests with post-hoc Bonferroni correction.

## Supplementary Material

Supplementary Files

This is a list of supplementary files associated with this preprint. Click to download.

- SupplementaryInformation.docx
- FigureS5.png
- FigureS1.png
- FigureS8.png
- FigureS7.png
- FigureS4.png
- FigureS6.png
- FigureS3.png
- FigureS9.png
- FigureS2.png
